# Recent Advances in Microenvironment-Responsive Materials for Periodontitis Therapy

**DOI:** 10.3390/ijms27114943

**Published:** 2026-05-29

**Authors:** Wenhan Ma, Yutong Han, Tong Cui, Jinfeng He, Haishan Shi

**Affiliations:** 1 School of Stomatology, Jinan University, Guangzhou 510632, China; 2 College of Chemistry and Materials Science, Jinan University, Guangzhou 511443, China; 3 Engineering Research Center of Artificial Organs and Materials, Ministry of Education, Jinan University, Guangzhou 511443, China

**Keywords:** periodontitis, pathological microenvironment, microenvironment-responsive materials, spatiotemporal orchestration, clinical translation

## Abstract

Periodontitis is a chronic inflammatory condition characterized by the progressive destruction of periodontal supporting tissues. With a global prevalence exceeding 60%, it poses a significant public health challenge. Traditional therapeutic approaches, primarily mechanical debridement, systemic antibiotics, and surgical interventions, often face limitations such as incomplete biofilm removal, rapid drug clearance, and systemic adverse effects. To overcome these challenges, recent research has shifted towards the development of intelligent biomaterials capable of modulating the pathological microenvironment. These microenvironment-responsive strategies leverage unique biochemical signatures, including acidic pH, elevated reactive oxygen species (ROS), and enzymatic dysregulation, to facilitate precise, on-demand drug delivery at the lesion site. This review examines recent advances from three integrated perspectives: (1) material platforms (hydrogels, microneedles, fiber membranes, microspheres, inorganic nanoparticles, and vesicles); (2) responsive design (pH, ROS, enzyme, glucose, and multi-stimulus cascade logic); and (3) spatiotemporal functional orchestration (early-stage microecological remodeling, mid-stage osteoimmunomodulation, and late-stage tissue regeneration). Additionally, we analyze critical translational challenges, including manufacturing scalability, clinical sterilization, and long-term biosafety, while discussing prospects for clinical implementation. This review aims to provide a strategic roadmap and theoretical guidance for the development of next-generation precision therapies for periodontitis.

## 1. Introduction

Periodontitis is a chronic inflammatory disease affecting the tooth-supporting structures. With a global prevalence exceeding 60%, it is a leading cause of tooth loss in adults [1,2,3]. Clinically, it manifests as gingival inflammation, deep periodontal pocket formation, and progressive destruction of alveolar bone and the periodontal ligament [2]. Periodontitis is classified into stages I–IV according to disease severity and into grades A–C according to the risk of disease progression, which ranges from low to high [4]. Traditional therapeutic approaches, including mechanical debridement, systemic antibiotics, anti-inflammatory agents, and surgery, aim to halt disease progression [5,6]. However, these conventional methods often face significant limitations. Mechanical scaling frequently fails to eliminate persistent biofilms harbored within anatomically complex pockets [7], while systemic antibiotic use carries risks of collateral dysbiosis and antimicrobial resistance [8]. Moreover, conventional local drug delivery is often compromised by rapid clearance due to continuous gingival crevicular fluid (GCF) flow [9]. These persistent clinical challenges highlight the urgent need for innovative, target-specific treatment strategies.

The periodontitis microenvironment represents not merely a localized bacterial infection but a spatiotemporally dynamic disruption of host osteoimmune homeostasis, driven by dysbiotic oral microbiota. Pathogenic microorganisms trigger the massive infiltration and activation of innate immune cells, particularly neutrophils and macrophages. These cells release pro-inflammatory cytokines, such as interleukin-1 (IL-1), tumor necrosis factor-α (TNF-α), and IL-6, and generate substantial amounts of reactive oxygen species (ROS) [10]. While these responses are initially essential for host defense, their excessive or chronic activation drives the local niche into severe oxidative stress. This sustains a state of hyperinflammation that upregulates matrix metalloproteinases (MMPs) and aberrantly activates osteoclasts, ultimately leading to irreversible tissue destruction [11,12]. Collectively, these pathological features, including acidic pH, elevated ROS, local hypoxia, and overexpression of specific enzymes and bacterial metabolites such as adenosine triphosphate (ATP), constitute a unique biochemical signature.

Rather than viewing this pathological microenvironment merely as a barrier to treatment, the biomaterials field has undergone a paradigm shift, leveraging these features as endogenous, disease-specific triggers for intelligent drug delivery. By engineering responsive carriers (including hydrogels, microneedles, fiber membranes, microspheres, inorganic nanoparticles, and vesicles) with intrinsic sense-and-respond logic, researchers can achieve on-demand, spatiotemporally orchestrated release of therapeutic agents. This approach significantly enhances local bioavailability, minimizes systemic side effects, and enables the synergistic execution of multifaceted functions, including antibacterial, immunomodulatory, antioxidant, and pro-regenerative activities.

**Figure 1:**
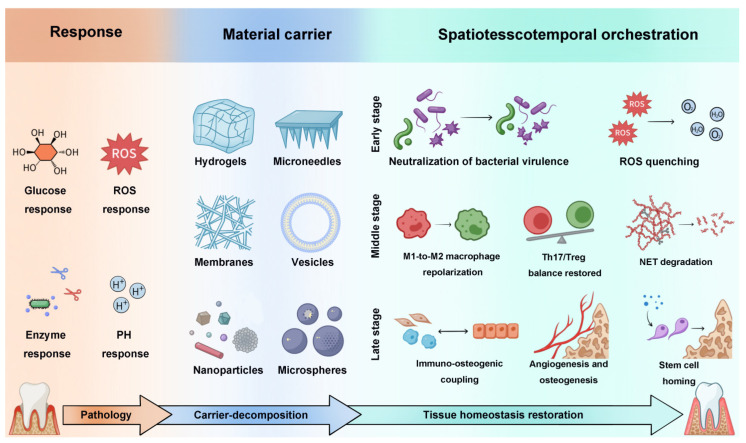
Strategic design of microenvironment-responsive platforms for periodontitis therapy. The framework integrates three dimensions: endogenous responsive design (detecting pH, ROS, MMPs, enzymes, glucose); carrier platforms (hydrogels, microneedles, fiber membranes, microspheres, inorganic nanoparticles, vesicles); and spatiotemporal functional orchestration across healing stages: early (neutralization of bacterial virulence, ROS quenching), mid (M2 polarization, NET degradation, Th17/Treg restoration), and late (angio-osteogenesis, stem cell homing, immuno-osteogenic coupling). The progressive arrow represents the causal chain from enhanced response signal-induced carrier decomposition to drug release and therapeutic effects, demonstrating the full process of converting pathological periodontitis into healthy periodontal tissue. The figure was created using Procreate Ver. 5.4.10.

In this review, we examine recent advances in microenvironment-responsive materials for periodontitis treatment, drawing on a comprehensive analysis of the PubMed and Web of Science databases. The search period spanned from the inception of each database to May 2026. The English search terms included “periodontitis, response, treatment, hydrogel, fiber membrane, microspheres, liposomes, inorganic nanoparticles, microneedles, exosomes”. Retrieved publication types comprised original research articles, reviews, commentaries, case reports, and meta-analyses. After removing articles that did not match the topic or were of low quality, supplemented by manual searching and the inclusion of seminal older literature, a total of 193 references were ultimately included in the review. Unlike conventional reviews that catalog material types or response mechanisms in isolation, we organize the literature around an integrated triad: material platforms, stimuli-responsive design, and spatiotemporal functional orchestration (Figure 1). We specifically highlight emerging conceptual frontiers such as multi-stimulus cascade responsiveness, osteoimmune reprogramming, and neutrophil extracellular trap (NET) intervention. Finally, we critically evaluate key translational barriers, from scalable manufacturing and clinical sterilization to long-term biosafety, that must be overcome to advance these platforms from bench-top prototypes to clinical realities.

## 2. Decoding the Periodontal Pathological Microenvironment: Endogenous Triggers for Responsive Design

**Figure 2:**
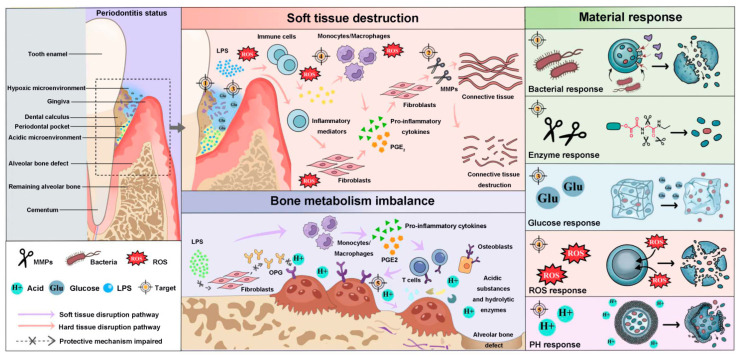
From pathological features to reactive chemistry. (**Upper left**) Schematic illustration of periodontitis. (**Bottom left**) Legend of symbols and annotations. (**Upper middle**) Mechanistic diagram depicting soft tissue damage induced by periodontal pockets. (**Middle and lower**) Mechanistic diagram depicting hard tissue damage induced by periodontal pockets. (**Right panel**) Schematic representation of the chemical paradigm for stimulus release, illustrating carrier deconstruction triggered by microenvironmental cues. The figure was created using Clip Studio Paint Ver. 4.0.

The pathogenesis of periodontitis is driven by oral microbiome dysbiosis, wherein pathogenic virulence factors and metabolic byproducts overwhelm host immune defenses, leading to chronic inflammation and tissue destruction [13]. This cascade results in a biochemical environment at the lesion site, characterized by localized acidosis, ROS accumulation, and elevated hydrolytic enzymes. Rather than viewing these solely as clinical challenges, microenvironment-responsive biomaterials exploit them as precise triggers for targeted drug delivery. We analyze key pathological signatures: acidic pH (Section 2.1), oxidative stress (Section 2.2), enzymatic dysregulation, encompassing host-derived MMPs and ALP as well as bacterial-secreted gingipains (Section 2.3), and hyperglycemia in diabetic periodontitis (Section 2.4). We conclude by discussing multi-stimulus cascade responsiveness (Section 2.5). Understanding these pathological cues establishes the foundation for functional material platforms (Section 3) and therapeutic orchestration (Section 4). Figure 2 shows the relevant content of this section.

### 2.1. Acidic Microenvironment and pH-Responsive Strategies

In periodontitis, persistent inflammation leads to metabolic byproduct accumulation and local hypoxia, creating an acidogenic environment [14,15]. During acute inflammation, the coexistence of deep periodontal pockets, severe gingivitis, or excessive host sugar intake can lower the pH within the periodontal pocket from approximately 7.4 to 5.5–6.5, providing a theoretical rationale for pH-responsive drug delivery systems [16]. Researchers have developed smart delivery platforms that remain stable under physiological conditions but disassemble upon reaching the acidogenic environment, enabling rapid, localized drug release. The design relies on two mechanisms: (i) protonation or deprotonation of ionizable groups [17] and (ii) cleavage of acid-labile covalent bonds [18]. The first strategy employs ionizable groups (e.g., amines) that undergo charge reversal upon protonation. For instance, Hu et al. [19] developed quaternary ammonium chitosan nanoparticles whose residual amines protonate at acidic pH, destabilizing the carrier and triggering doxycycline release. The second approach uses acid-labile linkages, including Schiff base and hydrazone bonds. A representative example is the hydrogel system reported by Li et al. [20], which employs a dynamic Schiff base network between quaternized chitosan and oxidized dextran. The imine bonds undergo selective hydrolysis in the acidic milieu, enabling synchronized antimicrobial and immunomodulatory agent release. Because localized acidosis frequently co-occurs with oxidative stress, integrating pH-sensitive with ROS-responsive elements represents a logical progression toward multi-stimulus interventions (Section 2.5). The pH-responsive design principles underlying these periodontal platforms are grounded in a broader body of polymer science. Kost and Langer [21] reviewed drug delivery applications throughout the body that are based on the response of polymer swelling to pH and ionic strength and demonstrated that altering the solution pH can induce polymer swelling or deswelling. Therefore, drug release from reservoirs or matrix devices composed of these polymers exhibits pH-dependent release kinetics, and the swelling process becomes more rapid when a larger proportion of the acid groups are in the non-ionized state.

### 2.2. Oxidative Stress and ROS-Responsive Strategies

Severe oxidative stress is a defining characteristic of the periodontitis microenvironment. Under physiological conditions, H_2_O_2_ is maintained below 1 µM [22,23]. During pathogenesis, hyperactivated infiltrating neutrophils undergo respiratory burst, generating excessive ROS (including superoxide anions, hydroxyl radicals, and H_2_O_2_) [24,25]. This persistent oxidative stress disrupts redox homeostasis, exacerbating matrix degradation and immune dysfunction [26,27]. In diabetic patients, hyperglycemia further intensifies ROS overproduction. Consequently, H_2_O_2_ concentrations in the inflamed periodontal niche have been reported to rise to approximately 50–200 µM in some studies, serving as a context-dependent trigger for targeted intervention [28].

ROS-responsive biomaterials exploit this elevation through oxidative cleavage of dynamic covalent bonds, particularly phenylboronic esters [29]. Phenylboronic esters undergo H_2_O_2_-triggered cleavage [30,31], a chemistry that has enabled advanced platforms for dual-action modulation. Gan et al. [32] employed a tri-thiol phenylboronic acid ester-crosslinked hydrogel to co-deliver macrophages and a C5a receptor antagonist; the network dissociates in the high-ROS niche, simultaneously mitigating oxidative stress and suppressing inflammation. The results demonstrated that the ROS-responsive hydrogels underwent rapid weight loss in a 1 mM H_2_O_2_ solution. When the H_2_O_2_ concentration was lowered to 100 μM to simulate the pathological periodontal microenvironment, the hydrogels gradually shrank, became transparent, and completely degraded within 120 h. In contrast, immersion in PBS buffer induced negligible changes in shape and size, confirming their ROS-responsive properties. Despite these promising in vitro results, the clinical translation of such autologous cell-loaded systems is primarily hindered by pronounced individual heterogeneity, the absence of long-term safety evaluations, persistent challenges in standardization, and the instability of drug release kinetics under dynamic in vivo conditions. Similarly, Zhao et al. [33] developed a dynamic Schiff base hydrogel for on-demand release of metformin and doxycycline in a diabetic periodontitis model. Coupling ROS-sensitive elements with pH- and enzyme-responsive mechanisms enables coordinated multi-stimulus therapy, as further elaborated in Section 2.5.

### 2.3. Enzymatic Dysregulation and Enzyme-Responsive Strategies

Beyond pH and ROS alterations, periodontitis is characterized by significant dysregulation of multiple enzymes, including matrix metalloproteinases, ALP, and bacterial-derived proteases such as gingipain. These enzymes function both as pathological drivers of tissue destruction and as highly specific endogenous triggers for smart drug delivery systems.

#### 2.3.1. Matrix Metalloproteinases (MMPs)

In periodontitis-affected tissues, neutrophils and macrophages secrete elevated levels of MMP-8 and MMP-9. During active disease, MMP levels correlate positively with pocket depth [34]. These enzymes directly degrade extracellular matrix components, including type I–V and type VII collagen. Additionally, they stimulate cytokine production that further promotes connective tissue degradation [35]. From a material design perspective, MMP-8 concentration in healthy GCF is below 10 ng/mL but often exceeds 100 ng/mL in periodontitis patients, correlating with pocket depth and disease severity [36,37]. This differential provides a distinct biochemical signal for enzyme-responsive delivery.

MMP-responsive biomaterials utilize these proteases to cleave specific peptide bonds or hydrolyze ester linkages. Peptide substrates containing MMP-2-cleavable sequences (e.g., GPLGVRG) are specifically recognized and cleaved, disrupting the carrier matrix and initiating drug release [38]. Xu et al. [12] developed an enzyme-responsive hydrogel incorporating copper-tannic acid nanosheets within a triglycerol monostearate matrix. As MMP levels rise, ester bonds are hydrolyzed, releasing butylated hydroxytoluene (BHT) for anti-inflammatory and antioxidative effects. Zhou et al. [38] fabricated an MMP-2-responsive hydrogel by crosslinking acetylated hyaluronic acid with an MMP-2-cleavable peptide; elevated MMP-2 activity in chronic periodontitis triggers hydrogel degradation and controlled drug release. In drug release experiments, the synthetic hydrogel released slowly in collagenase-free medium, reaching 42% cumulative release after 21 days. In the presence of 5 U/mL collagenase, release was faster and more sustained, attaining 71% cumulative release at 21 days—1.7-fold higher than that observed without collagenase. This pronounced enhancement indicates a significant collagenase-responsive drug release behavior. Despite these promising release profiles, the clinical application of MMP-responsive hydrogels remains hampered by the inherent hydrophobicity and potential off-target toxicity of encapsulated agents such as GSK2606414, underscoring the need for further carrier optimization before clinical translation. Combining MMP sensitivity with pH and ROS responses is discussed in Section 2.5.

#### 2.3.2. Alkaline Phosphatase (ALP)

Beyond host-derived MMPs, ALP has emerged as a reliable diagnostic biomarker for periodontal disease progression. ALP is produced by various cells and bacteria within periodontal pockets, and its elevated activity correlates with disease severity [39]. ALP levels in GCF are significantly elevated in patients with chronic periodontitis compared with healthy individuals. In one study, a specific healthy population meeting the predefined inclusion criteria exhibited a GCF ALP level of approximately 776.76 ± 121.91 IU/L, whereas the level in chronic periodontitis patients reached approximately 1825.77 ± 275.12 IU/L (*p* < 0.001) [40]. The ability of ALP to hydrolyze phosphoester bonds under physiological conditions offers a unique mechanism for designing enzyme-responsive materials. ALP catalyzes nucleophilic attack on the phosphate group, cleaving P-O-P bonds and disrupting the carrier matrix [41]. Exploiting this mechanism, Li et al. [42] developed an ALP-responsive membrane for periodontitis treatment. A chitosan membrane loaded with minocycline hydrochloride was crosslinked with polyphosphoesters. As periodontitis progresses, ALP secreted into the periodontal pocket hydrolyzes the polyphosphoester crosslinks, promoting polymer degradation and sustained, site-specific minocycline release. ALP levels remain low in healthy gingiva, ensuring minimal off-target release. In vitro release assays showed that the cumulative release of minocycline hydrochloride from the fibrous membrane was below 60% without ALP but increased to approximately 80% upon ALP exposure, verifying the enzyme-responsive behavior of the material. Nevertheless, translating ALP-responsive membranes into clinical practice will require systematic evaluation of long-term degradation product safety and biocompatibility, alongside resolution of manufacturing scalability and sterilization compatibility challenges.

#### 2.3.3. Gingipain: Toward Virulence Disarmament

Whereas MMP- and ALP-responsive systems react to host-derived inflammatory markers, targeting pathogen-derived virulence factors represents a shift toward precision microbiome modulation. Oral microbial dysbiosis initiates periodontitis [43]. *Porphyromonas gingivalis*, a keystone pathogen, secretes gingipains, cysteine proteases (RgpA, RgpB, Kgp) that enable tissue invasion, degrade host proteins, and orchestrate immune evasion [44,45]. Concurrently, these proteases activate host defense cells and induce inflammatory mediator release, leading to secondary tissue damage.

The pathogen-restricted expression of gingipains makes them highly specific triggers for on-demand virulence disarmament. Gingipain activity is minimal in healthy aerobic gingival sulci, where the symbiotic microenvironment inhibits *P. gingivalis* colonization. Conversely, the hypoxic periodontal pocket facilitates robust *P. gingivalis* proliferation and drastic upregulation of gingipains [46]. Consequently, elevated GCF gingipain levels correlate strongly with clinical severity parameters, including probing depth (PD), clinical attachment loss (CAL), and bleeding on probing (BOP) [46]. Liu et al. [47] developed a gingipain-responsive hydrogel that releases antibacterial and regenerative agents upon exposure to *P. gingivalis*. The hydrogel, synthesized via Michael addition, contains a short antimicrobial peptide flanked by gingipain-cleavable domains. Upon contact with *P. gingivalis*, secreted gingipains cleave these anchor domains, liberating the antimicrobial peptide for localized antibacterial action. Concurrently, the degrading hydrogel releases stromal cell-derived factor-1 (SDF-1), facilitating host stem cell recruitment and osteogenic differentiation. This dual-action design exemplifies the potential of using pathogen-specific enzymes to eradicate keystone pathogens while preserving commensal flora.

### 2.4. Hyperglycemia and Glucose-Responsive Strategies in Diabetic Periodontitis

Diabetes mellitus and periodontitis exhibit a bidirectional relationship, with diabetic individuals facing a threefold increased risk of severe periodontal destruction [48]. In a specific subset of patients with both diabetes and periodontal disease, the glucose concentration in gingival crevicular fluid (GCF) can be markedly elevated from <5.0 mM, a level typically observed in healthy individuals, to an approximate range of 7.0–10.0 mM, as documented in several studies [49]. This hyperglycemic environment not only exacerbates oxidative stress and advanced glycation end product (AGE) accumulation but also serves as a disease-specific biochemical trigger.

Two principal chemical strategies exploit this elevated glucose. The first employs glucose oxidase (GOx), which catalyzes glucose oxidation to gluconic acid and H_2_O_2_, generating dual triggers, a pH decrease and ROS elevation, that initiate degradation of pH- or ROS-sensitive carriers [50]. Tang et al. [50] developed a hydrogel system comprising a GOx/peroxidase/minocycline-loaded zeolitic imidazolate framework-8 (ZIF-8) core encased in a calcium alginate shell. The hydrogel system exhibits glucose-responsive behavior. Under high-glucose conditions, gluconic acid generated by glucose oxidase dissolves the acid-sensitive ZIF-8 framework, thereby triggering the release of therapeutic agents and alleviating inflammatory responses in diabetic models. In vitro release studies showed that after 90 min of exposure to 5 mM glucose, the cumulative release of minocycline reached 25%. In contrast, at glucose concentrations of 15 mM and 35 mM, more than 50% and 75% of minocycline were released within the same period, respectively, confirming the glucose-dependent release profile. Nevertheless, the clinical translation of GOx-based glucose-responsive systems faces inherent challenges: the enzymatic activity of GOx may be compromised during sterilization or long-term storage, degradation products of the ZIF-8 framework require systematic biocompatibility evaluation, and the complexity of the multi-component system poses significant hurdles for scalable manufacturing and quality control.

The second strategy leverages enzyme-free dynamic phenylboronic acid (PBA) chemistry [51]. PBA binding with 1,2- or 1,3-diols in glucose converts the boron center from a neutral trigonal planar geometry to a hydrophilic tetrahedral boronate ester, accelerating hydrogel swelling and payload diffusion. Feng et al. [52] crosslinked PBA-functionalized alginate with carboxymethyl chitosan to deliver epigallocatechin gallate (EGCG). As glucose rises to diabetic levels, dynamic boronate bonds dissociate, triggering on-demand EGCG release with anti-inflammatory and anti-resorptive effects. Threshold matching (stability at glucose < 5.0 mM and degradation at >7.0 mM) and integration with pH/ROS responses are discussed in Section 2.5. Furthermore, glucose-responsive designs have also demonstrated important applications in non-periodontal fields. Tanna et al. [53] fabricated glucose-responsive materials for insulin delivery by employing dextran methacrylate (dex-MA) and concanavalin A methacrylamide (Con A-MA) as the constituent raw materials. In the absence of free glucose, the binding sites on Con A recognize and bind to the glucose residues along the dex-MA molecular chains. When free glucose appears in the environment, these small molecules compete with the glucose residues on the dex-MA chains for the Con A binding sites. Owing to the higher binding affinity of free glucose, the physical crosslinking points dissociate, the gel network collapses, and a glucose-triggered response is thereby achieved.

### 2.5. Multi-Stimulus-Responsive Strategies: Spatiotemporal Synchronization

The periodontal microenvironment features concurrent and sequential fluctuations of pH, ROS, enzymes, and glucose. Single-stimulus systems may cause false positives (off-target release) or false negatives (insufficient dosing) [54,55]. To enhance precision, biomaterial design is advancing toward multi-responsive cascade programming, guided by three principles: temporal cascading, conditional gating, and spatial heterogeneity.

Temporal cascading exploits the natural sequence of pathological events. In periodontitis, acute inflammation and enzymatic overexpression typically precede sustained acidification [56,57]. Hierarchical materials can be engineered such that an initial stimulus unlocks the outer protective layer, exposing the inner core to a secondary trigger. For example, a core–shell microneedle patch with dual MMP/pH responsiveness has been developed: the MMP-sensitive outer shell degrades rapidly upon encountering elevated enzymes, delivering an antibacterial burst, while the inner core subsequently disassembles under acidic pH to release osteogenic factors [58].

Conditional gating requires two or more stimuli to trigger release, functioning as a Boolean AND logic gate. A hydrogel crosslinked with both pH-labile (Schiff base) and ROS-labile (phenylboronic ester) bonds remains intact unless both acidosis and elevated ROS are present simultaneously, ensuring payload liberation only in the fully activated pathological niche [59].

Spatial heterogeneity addresses the complex anatomical interface of the periodontal pocket, where pH, enzyme activity, and bacterial density vary from the superficial gingival margin to the deep alveolar bone defect. A spatially synergistic system can be engineered with a multi-compartment architecture; for instance, a scaffold with a gingipain-responsive outer layer for biofilm eradication and an MMP-responsive inner core for guided bone regeneration [60]. Table 1 summarizes the responsive mechanisms and their threshold alignment. The following sections examine how these designs achieve spatiotemporal orchestration, from early microecological restoration to late-stage tissue regeneration.

## 3. Material Platforms and Clinical Adaptability: From Passive Carriers to Active Modulators

**Figure 3:**
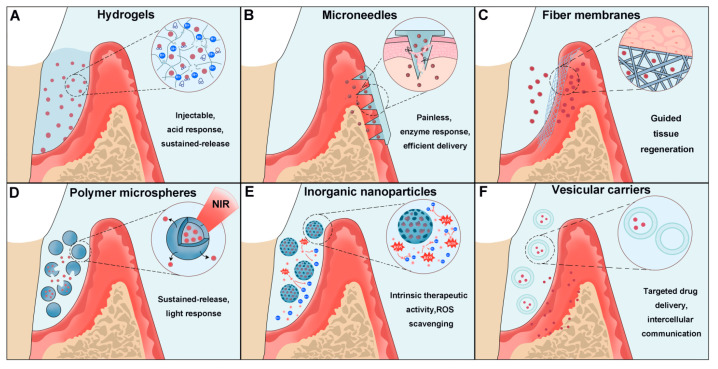
Microenvironment-responsive carrier platforms for periodontitis therapy. (**A**) Hydrogels: injectable, conformable, spatiotemporally locked depots triggered by acidosis. (**B**) Microneedles: painlessly bypass epithelial barrier for deep-tissue delivery. (**C**) Fiber membranes: asymmetric guided tissue regeneration (GTR) barriers excluding epithelial cells while guiding tissue regeneration. (**D**) Polymer microspheres: adhesive, intracellular-targeting depots for prolonged release. (**E**) Inorganic nanoparticles: rigid carrier-is-drug platforms with nanozyme activity and theranostic functions. (**F**) Vesicles (exosomes, liposomes): biomimetic carriers enabling membrane fusion and cell-free signaling. The figure was created using Clip Studio Paint Ver. 4.0.

Given the intricate topography of periodontal lesions, the selection of carrier platforms is as crucial as the therapeutic agents they convey. This section critically assesses the clinical adaptability and translational limitations of various microenvironment-responsive platforms, as summarized in Table 2 and Figure 3.

### 3.1. Hydrogels: Injectable Drug Depots for Deep Periodontal Pockets

Hydrogels are injectable and conformable to deep pockets, with precursors that undergo in situ gelation to fill complex defects [74,75]. Their high water content effectively protects biomacromolecules, including proteins, nucleic acids, and antibodies, from denaturation [76]. Accordingly, hydrogels serve as versatile carriers for small molecules, proteins, growth factors, and other biologics [77]. Moreover, their tunable degradation rates, controllable physicochemical properties, and relatively straightforward fabrication [78] make them promising platforms for stage-specific periodontitis treatment. However, their weak mechanical strength and high swelling ratios render them susceptible to washout by GCF flow and masticatory forces [79]. To address this, modern hydrogels incorporate stimulus-cleavable crosslinks (e.g., Schiff base, boronate ester) that remain stable under physiological conditions but degrade selectively during inflammation, achieving spatiotemporally controlled drug retention. For instance, Cai et al. [63] developed a Schiff base hydrogel that releases embelin under acidic pH to promote osteoimmunomodulation.

### 3.2. Microneedles: Bypass the Epithelial Barrier via Compartmentalized Delivery

Microneedles (MNs) painlessly penetrate the keratinized gingival epithelium, enabling drug delivery into the underlying connective tissue [80,81,82]. In the oral environment, MNs face challenges from salivary flow and mechanical forces, which are mitigated by bio-inspired wet-adhesion backings or swellable matrices [83,84]. Li et al. [64] developed a photo-crosslinkable MN patch with wet-adhesion capability. Shan et al. [65] developed a core–shell MN patch in which palladium nanozymes in a dissolving core provide an antibacterial burst, while cerium nanozymes in a durable shell enable sustained NET degradation. MN platforms complement hydrogels by bypassing the epithelial barrier while offering tunable release kinetics.

### 3.3. Fiber Membranes: Physical Barriers with Biofunctional Interfaces

Guided tissue regeneration (GTR) membranes prevent epithelial downgrowth and support periodontal regeneration [85,86,87]. Composed of polymers with high specific surface area and porosity, these fibrous membranes are well suited for drug loading and sustained release and have been extensively employed in periodontal regeneration [88,89]. Recent designs focus on biofunctional interfaces. Han et al. [66] fabricated a polycaprolactone/poly(lactic-co-glycolic acid) (PCL/PLGA) membrane incorporating tea polyphenol-functionalized graphene oxide to promote M2 macrophage polarization. Another approach uses asymmetric (Janus) architectures. Cheng et al. [67] designed a double-layered piezoelectric poly-L-lactic acid (PLLA)-zinc oxide membrane: the soft-tissue side blocks epithelial ingrowth, while the bone-facing side generates electrical microenvironments under masticatory stress, activating Piezo1 channels and downstream osteogenic signaling. The results show that NCTC clone 929 cells failed to penetrate the membrane within 24 h, confirming that the membrane effectively blocks fibroblast infiltration and thus prevents soft tissue outgrowth toward the bone resorption site. The bilayer architecture further reinforced the barrier function of the composite membrane. Thus, fiber membranes can serve as both passive barriers and active mechanobiological interfaces.

### 3.4. Polymer Microspheres: From Tissue Adhesion to Intracellular Modulation

Microspheres provide sustained release over weeks to months [90,91]. However, conventional microspheres relying on passive entrapment are rapidly cleared by GCF [90,92]. Modern designs focus on surface functionalization and intracellular targeting. Xiu et al. [68] employed polydopamine-coated microspheres for mucosal adhesion and photothermal antibacterial effects. The results demonstrated that the microspheres exhibited excellent adhesion to human-derived in vitro teeth, as well as to metal, glass, and plastic surfaces. Moreover, even after flipping and twisting, the microspheres remained well adhered to rat maxillary gingival tissue in vitro. The microspheres also possessed favorable photothermal conversion performance. Under near-infrared light at an intensity of 1 W/cm^2^, the temperature of the microspheres increased from 26.5 °C to 55 °C within approximately 6 min. At a laser intensity of 0.5 W/cm^2^, the maximum temperature reached 45 °C. At a laser intensity of 1.5 W/cm^2^, the temperature rapidly rose to about 60 °C within 2 min and ultimately reached approximately 67 °C. In addition, their micro- and nano-scale dimensions enable endosomal escape and intracellular ROS scavenging, reprogramming macrophages toward the M2 phenotype. Ming et al. [69] developed sericin/alginate microspheres loaded with proanthocyanidins to promote biomineralization and attenuate oxidative stress. Thus, microspheres combine extracellular adhesion with intracellular modulation for prolonged multi-target action.

### 3.5. Inorganic Nanoparticles: Multimodal Theranostic Nanoplatforms

Inorganic nanoparticles offer rigid architectures, high surface-area-to-volume ratios, and inherent bioactivity [93]. Key capabilities include nanozyme-mediated catalysis, rigid mesoporous confinement for gene delivery, and theranostic integration. Cerium oxide nanoparticles mimic superoxide dismutase and catalase [94], while silver nanoparticles exhibit broad-spectrum antibacterial activity [95]. This carrier-is-drug paradigm reduces reliance on conventional antibiotics. Unlike soft carriers, rigid inorganic frameworks protect nucleic acid payloads from enzymatic degradation [70]. Gold, mesoporous silica, and iron oxide nanoparticles enable fluorescence, photothermal, or magnetic resonance imaging for real-time therapeutic monitoring [96,97]. In addition, inorganic nanoparticles exploit both the enhanced permeability and retention (EPR) effect and the extravasation through leaky vasculature and inflammatory cell-mediated sequestration (ELVIS)-like effect [98,99]. The EPR effect represents the principal mechanism for passive tumor enrichment, predominantly in solid tumors: the structurally incomplete neovasculature formed during rapid tumor growth, characterized by wide interendothelial gaps, permits the extravasation of appropriately sized nanoparticles, which then selectively accumulate at high concentrations within the tumor tissue; concomitantly, impaired lymphatic drainage hinders the removal of the infiltrated nanoparticles, thereby enabling prolonged intratumoral retention. The ELVIS effect constitutes a passive targeting mechanism by which nanomedicines home to inflammatory tissues. Analogous to tumors, inflammatory sites exhibit elevated vascular permeability that allows nanoparticle extravasation; subsequently, the extravasated nanoparticles are actively internalized by the abundant inflammatory cells at the site, resulting in rapid cellular uptake and intracellular processing.

Li et al. [70] employed hollow copper oxide (Cu_x_O) nanoparticles for miRNA-126 delivery and enzyme-mimetic catalysis; their degradation releases copper ions that further contribute to immunomodulation. The results demonstrated that treatment with this nanoparticle significantly reduced the expression of IL-8, IL-6, IL-1β, and iNOS in the LPS-treated group, indicating a potent anti-inflammatory effect. In RAW264.7 cells, it also increased the number of Arg-1-positive cells (anti-inflammatory phenotype) and decreased the number of iNOS-positive cells (pro-inflammatory phenotype), highlighting its role in promoting macrophage polarization toward an anti-inflammatory phenotype. Yang et al. [71] developed quercetin-loaded mesoporous bioactive glass (MBG) nanoparticles, in which the MBG matrix releases osteostimulatory ions (Si, Ca) while quercetin provides immunomodulation. Inorganic nanoparticles thus transcend passive delivery, offering theranostic capabilities and potential for personalized adaptation.

### 3.6. Vesicular Carriers: Biomimetic Membrane Fusion and Cell-Free Signaling

Vesicles enable direct membrane fusion with target cells [100] and encompass naturally derived exosomes and synthetic liposomes. Exosomes exhibit minimal immunogenicity. Qiao et al. [72] demonstrated that dental pulp stem cell (DPSC)-derived exosomes promote M1-to-M2 macrophage polarization and osteogenesis. Liposomes are synthetic spherical vesicles composed of one or more phospholipid bilayers, ranging from 30 nm to several micrometers in diameter. Their biodegradability, biocompatibility, and capacity to protect labile drugs make them attractive carriers [101]. Liposomes and exosomes likewise exhibit both EPR and ELVIS effects [102,103,104,105]. Owing to the high permeability of tumor vasculature and impaired lymphatic drainage, long-circulating liposomes repeatedly perfuse the tumor region via the bloodstream, extravasate through the gaps between leaky vessels, and become entrapped as a result of obstructed lymphatic return. The extravasation of exosomes depends not solely on passive extrusion through interendothelial gaps but may also involve active transcellular transport across the endothelial barrier, thereby enabling more effective execution of their functions. Within inflammatory lesions, liposomes, as foreign particulates, inherently serve as both targets and drug reservoirs. The complex natural membrane protein repertoire of exosomes permits more efficient and specific interactions with inflammatory cells. However, liposomes are structurally fragile and can be stabilized by embedding them in hydrogel composites. Atila et al. [73] developed an injectable liposome-in-hydrogel composite (curcumin and α-tocopherol encapsulated in liposomes embedded within a pH-sensitive chitosan hydrogel), achieving sustained release for up to 14 days. Vesicular platforms thus bridge synthetic design and biological communication (see Table 2).

### 3.7. Characteristics and Differences in Different Carrier Materials

Hydrogels, microneedles, and fibrous membranes function as macro-scale delivery carriers in periodontitis treatment, sharing drug-loading capacity, programmed sequential release, and the ability to integrate with nanomaterials for synergistic theranostics. Their principal differences, however, lie in design rationale and application modality: injectable hydrogels are delivered as fluids into periodontal pockets, acting as three-dimensional scaffolds with minimal mechanical requirements, making them particularly suited for filling deep, irregular osseous defects and delivering biologics that require protection from enzymatic degradation [106]; microneedle patches penetrate the gingival epithelium to target deep lesions, demanding strong adhesion and sufficient needle-tip mechanics, rendering them most applicable when transepithelial drug penetration into the inflamed connective tissue is the primary therapeutic goal [83]; fibrous membranes are applied as two-dimensional barrier covers that isolate the defect, requiring high flexibility, adequate tensile strength, and a degradation timeline matched to guided bone regeneration, positioning them as the platform of choice for GTR procedures involving alveolar bone defects [107].

Inorganic nanoparticles, polymer microspheres, liposomes, and exosomes all represent micro/nanoscale drug-loaded particles that share high specific surface area, high drug-loading capacity, and the ability to encapsulate, adsorb, or solubilize labile small-molecule drugs, proteins, and nucleic acids; their surfaces further permit chemical or biological modification for modular integration of targeting, therapeutic, and imaging functionalities. Their distinctions, however, are pronounced across origin, drug-loading mechanism, stability, and functionality. Polymer microspheres, inorganic nanoparticles, and liposomes are synthetically derived, whereas extracellular vesicles are natural biological particles. Drug loading occurs through fundamentally different modes: polymer microspheres entrap drugs within dense polymer segment networks; inorganic nanoparticles are impermeable rigid crystal cores that load cargo solely via surface coordination, electrostatic adsorption, or encapsulation in engineered mesopores without lattice involvement; liposomes are artificial self-assembled phospholipid bilayers that localize drugs based on hydrophilicity/hydrophobicity; and extracellular vesicles are naturally secreted biomembrane vesicles into which microRNA, proteins, and lipids are actively sorted and integrated. Liposomes and exosomes exhibit inferior stability and are more difficult to store long-term relative to inorganic nanoparticles and polymer microspheres [108,109]. Functionally, inorganic nanoparticles stand out due to their intrinsic fluorescence, photothermal conversion, and anti-inflammatory/antibacterial properties [110,111], though high concentrations may provoke strong immune responses or cytotoxicity [112], and their carrier-is-drug functionality makes them uniquely valuable when catalytic ROS scavenging or real-time theranostic monitoring is required alongside drug delivery; polymer microspheres provide excellent bioinertness and high loading but weak biological interactions, making them best suited for scenarios requiring prolonged, site-specific drug retention over weeks to months with minimal systemic exposure [113]; liposomes reduce toxicity by mimicking biological membranes and can directly fuse with cell membranes for cargo release, demonstrating good biocompatibility [114]; and exosomes carry a multidimensional repertoire of transcriptomic and proteomic information capable of regulating recipient cell expression, offering unique bioactive advantages that are particularly relevant for immunomodulation and stem cell-mediated regeneration in the late healing phase [115].

### 3.8. Microenvironment-Responsive Materials Versus Traditional Treatments: Clinical Benefits, Limitations, and Translational Potential

Conventional pharmacotherapy suffers from inherent shortcomings, including insufficient local drug concentration, short retention time, and the difficulty of integrating antibacterial, anti-inflammatory, and pro-regenerative functions into a unified therapeutic system [116]. The core advantage of microenvironment-responsive materials resides in their ability to sense characteristic biochemical markers within the local pathological milieu of periodontitis and, through pre-programmed physical or chemical transitions, trigger the execution of therapeutic functions at the appropriate time and site. This capability enables on-demand release, prolonged drug retention, extended duration of action, and markedly improved drug utilization. Simultaneously, multifunctional integrated therapy can be achieved by co-loading drugs or particulate components with distinct functionalities. For instance, in contrast to traditional local drug delivery into the periodontal pocket, hydrogel-based materials can be directly injected into the pocket to undergo in situ gelation and subsequently degrade to release therapeutics on demand in proportion to the severity of local inflammation. This mechanism circumvents the typical predicament of conventional sustained-release formulations, excessively high initial drug concentration followed by subtherapeutic late-stage levels, thereby maintaining the local drug concentration within an efficacious and safe therapeutic window throughout the entire treatment period [117]. Furthermore, multi-stage therapeutic execution with temporally orchestrated regulation can be realized through hierarchical structural design or the integration of functional modules possessing distinct response thresholds within a single hydrogel system [118].

Nevertheless, the translation of microenvironment-responsive materials from laboratory settings to clinical practice confronts a series of profound challenges, which originate from both the inherent constraints of the material design paradigm and the unique complexity of the oral physiological environment. These challenges encompass the unpredictability of material behavior in heterogeneous microenvironments, unresolved issues concerning the safety and long-term toxicity of therapeutic actions, industrial bottlenecks in manufacturing standardization and quality control, and a critical paucity of human validation studies. Despite these hurdles, the clinical translation and eventual application of microenvironment-responsive materials retain considerable promise: the manufacturing processes, production standards, and quality control protocols for hydrogels and microneedle patches are progressively maturing, and numerous drug-loaded hydrogels and microneedle systems have already entered early-stage clinical trials for periodontal therapy [83]; nanozymes, which function independently of chemical dosage and circumvent conventional drug-resistance mechanisms, offer distinct prospects for clinical adoption [119]; and advances in exosome production technology, declining manufacturing costs, and increasingly sophisticated standardized characterization of their active constituents collectively position extracellular vesicles as compelling candidates for clinical investigation in periodontal regenerative therapy. Collectively, these indicators suggest that microenvironment-responsive biomaterials possess tangible feasibility for near-future application in the clinical management of periodontitis.

### 3.9. Manufacturing and Supervision of Materials

The large-scale translation of these six materials faces intertwined production, manufacturing, and regulatory challenges. Hydrogels allow fine-tuning of mechanical properties, degradation, and drug release via polymer and crosslinking selection and offer injectability for in situ gelation and minimally invasive delivery. However, scaling up is difficult because maintaining batch consistency in crosslinking density, porosity, and network architecture is challenging, and uneven temperature and shear forces cause inter-batch variability; mechanical performance, sterilization, and storage further hinder clinical use. Regulatory hurdles arise because hydrogel performance depends on multidimensional parameters (crosslinking, swelling, rheology, porosity, degradation) lacking standardized definitions and testing guidance. Microneedle manufacturing is relatively mature, with five established types selectable by drug and clinical needs, but ensuring consistent height, shape, sharpness, and strength across large batches remains difficult, and tip geometry quality control directly impacts penetration and delivery accuracy [120]. Regulatory gaps include the absence of dedicated oral microneedle standards and non-standardized testing for key indicators (fracture force, insertion force, drug uniformity), which impedes cross-study comparison; the need to simultaneously validate penetration, drug release kinetics, and clinical efficacy also makes trial design more complex than for conventional topicals. Industrial production of mesoporous silica and metal oxide nanoparticles is comparatively mature, and sol–gel methods can support scale-up [121], yet batch consistency and costly purification persist. Safety concerns exist because nanoparticles may enter the circulation through compromised pocket epithelium, and although ISO/TR 10993-22 guides nanomaterial biological evaluation [122], standards for long-term toxicity, immunogenicity, and clearance remain incomplete, with no unified global nanomedicine assessment framework.

The manufacturing of polymer microspheres and fibrous membranes is well established [123,124], yet microspheres still face challenges in particle size control, batch reproducibility, and cost, while fibrous membranes struggle to match mechanical properties and degradation to tissue regeneration dynamics, and processes like electrospinning are highly sensitive to temperature and humidity. From a regulatory perspective, drug-loaded microspheres may be classified as drugs or combination products, and drug release in the periodontal pocket is influenced by enzymatic degradation, pH fluctuations, and mechanical forces, complicating the development of reliable in vitro release models; in contrast, guided tissue regeneration membranes are Class III implantable devices with a dedicated ISO 22803:2004 standard and thus have a relatively mature regulatory pathway [125]. Extracellular vesicles and liposomes are reasonably mature at a laboratory scale, but both suffer from poor stability and high production costs. Liposomes are clearly categorized as nanomedicine delivery systems, and regulators have progressively raised evaluation standards, with the lack of long-term stability data remaining a major translational barrier. Extracellular vesicles, as acellular yet bioactive nanoscale vesicles, are increasingly regarded by the FDA as biologics or advanced therapeutic medicinal products, which imposes stringent requirements on manufacturing and quality control [126]; moreover, their pronounced compositional and functional heterogeneity across tissue sources makes establishing stable, renewable cell sources and corresponding quality standards a foremost challenge.

## 4. Spatiotemporal Orchestration of Periodontal Regeneration

**Figure 4:**
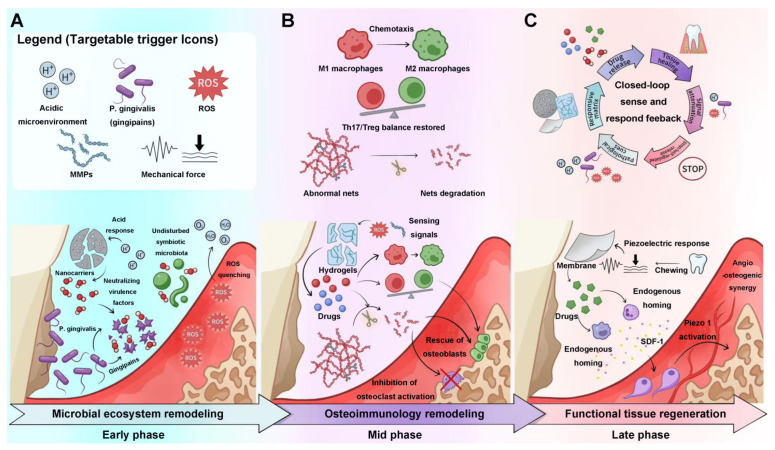
Spatiotemporal coordination of microenvironment-responsive functions. The system transitions with the disease timeline, detecting triggers (pH, gingipains, ROS, MMPs, mechanical force). (**A**) Early stage: virulence disarmament. (**B**) Mid-stage: M2 polarization, Th17/Treg restoration, NET degradation via nanozymes. (**C**) Late stage: pro-regenerative scaffold with stem cell homing, angio-osteogenic synergy. All-in-one logic: in response to signal enhancement, the carrier degrades to release the drug; periodontal tissue then recovers health, pathological signals weaken, and drug release ceases. The figure was created using Procreate Ver. 5.4.10.

The periodontitis microenvironment evolves through sequential stages, from microbial dysbiosis to chronic tissue destruction. Single-function or bolus-release strategies are poorly suited to this dynamic progression. Responsive materials (Section 2) must evolve into spatiotemporal orchestrators, executing three sequential functions: (i) early microecological remodeling, (ii) mid-stage osteoimmunomodulation, and (iii) late-stage tissue regeneration. The following subsections elaborate on these phases (summarized in Table 3 and Figure 4).

### 4.1. Early-Stage Microecological Remodeling: Virulence Disarmament and ROS Quenching

Periodontitis is driven by dysbiotic biofilms. Traditional broad-spectrum biocides harm commensal flora and promote resistance. An emerging approach focuses on microecological remodeling, disarming virulence rather than indiscriminately killing bacteria [142]. Key mechanisms include the following:

- Virulence factor neutralization: inhibitors or peptides that block *P. gingivalis* gingipains [143,144]. The strategy of neutralizing virulence factors, exemplified by the inhibition of *Porphyromonas gingivalis*, has received strong support from preclinical studies, with numerous experiments consistently demonstrating therapeutic benefits in preclinical models [145]. Nevertheless, the paucity of clinical research underscores the urgent need for further development and clinical evaluation.
- Biofilm matrix disruption: enzymatic degradation of extracellular polymeric substances (EPS) to dismantle biofilm architecture [146]. Non-fungicidal matrix-degrading enzymes represent a promising biofilm control strategy, offering the advantage of disrupting the EPS matrix while preserving the oral symbiotic microbiota. However, owing to their narrow substrate specificity and the inherent mismatch with the complex structural diversity of EPS polysaccharides, proteins, and eDNA, this approach has only been validated in vitro, with no clinical trial reports available for periodontitis patients.
- Quorum sensing interference: enzymatic degradation of autoinducers to disrupt bacterial communication [147,148]. A study published in 2025 confirmed that the AHL lactonase Est816 significantly reduces the biomass and thickness of periodontal pathogenic biofilms and alters the microbial community structure [149]. However, this study was based on in vitro cultures of subgingival plaque samples obtained from 30 patients with stage III or higher periodontitis and did not constitute an in vivo clinical investigation. Another study evaluated the efficacy of Est816 in combination with antibiotics using a rat model of periodontitis [147]; although promising, this work remains at the animal experimental stage and has not yet advanced to clinical practice.
- LPS neutralization: cationic polymers that adsorb lipopolysaccharide (LPS), blocking downstream inflammation [146]. The strategy of neutralizing LPS using cationic polymers has shown promise in both in vitro and animal experiments. In a recent study, cationic polymers effectively captured molecular patterns associated with anionic microorganisms, including LPS and cell-free DNA (cfDNA), and significantly reduced inflammatory alveolar bone loss in a ligature-induced mouse model of periodontitis, without causing notable adverse effects on the oral mucosal microbiome [150]. Nevertheless, the findings are currently limited to animal studies.

Responsive platforms apply these principles. For example, hydrogels that release antimicrobial peptides upon gingipain cleavage selectively suppress pathogens while sparing commensal flora [47]. Additionally, the pathogen-triggered oxidative burst can be quenched by ROS-scavenging interventions employing antioxidants [47,133,151,152]. Traditional antioxidant strategies rely on small-molecule antioxidants that are consumed during ROS neutralization, yielding only transient effects. In contrast, catalytic nanomaterials (nanozymes) function as recyclable catalysts, achieving sustained ROS scavenging without self-consumption, thereby overcoming the limitations of conventional antioxidant therapies [153,154]. By neutralizing virulence and restoring redox homeostasis, these interventions halt acute tissue degradation and create a permissive state for mid-stage immunomodulation.

### 4.2. Mid-Stage Osteoimmunomodulation: Resolving Inflammation and Restoring Immune Homeostasis

Following initial remodeling, the periodontal microenvironment enters a prolonged inflammatory phase characterized by M1-skewed macrophages, aberrant NET formation, and an elevated Th17/Treg ratio [10,11,155,156]. Mid-stage intervention requires sustained osteoimmunomodulatory strategies to reprogram the host immune-skeletal axis.

#### 4.2.1. Macrophage Polarization with Sustained Catalytic Antioxidants

Chronic stimulation polarizes macrophages toward the M1 phenotype, exacerbating tissue destruction [155]. Repolarization toward the M2 phenotype is a key therapeutic target. Advanced platforms employ nanozymes with sustained catalytic activity. For example, Xu et al. [12] developed a hydrogel incorporating copper-based nanozymes that provide continuous ROS scavenging and promote M2 polarization. Additionally, materials can activate the Nrf2 pathway [157] or deliver small-molecule modulators such as 8-aminoguanidine to suppress M1 activation [132]. Controlled release of magnesium ions (Mg^2+^) also promotes M2 polarization [158]. The application of nanozyme-based antioxidants remains at the preclinical stage, with relevant studies validated in vitro or in rat models of periodontitis. Although no clinical research has been conducted on periodontitis patients, clinical progress has been made in the treatment of periapical periodontitis using nanozymes, providing key evidence for their clinical translation in oral infectious diseases. The induction of Nrf2 pathway activation for periodontitis treatment is still in early clinical exploration, with existing studies primarily being observational or associational and focused on mechanistic validation; definitive confirmation is lacking. The delivery of small-molecule modulators or metal ions is currently at the in vitro validation stage and remains far from clinical application.

#### 4.2.2. Neutrophil and Nets Intervention: Dismantling Persistent Damage-Associated Molecular Patterns

Excessive ROS trigger NET formation [159]. Innovative platforms prevent or dismantle NETs. Zhuo et al. [130] employed exosomes derived from apoptotic neutrophils to induce neutrophil apoptosis and prevent NET release. For pre-existing NETs, Shan et al. [65] developed a microneedle system incorporating cerium nanozymes capable of degrading NET structures. To date, no clinical studies on periodontitis have directly targeted the induction of neutrophil apoptosis or the prevention of NET release. Current evidence is limited to correlating NET levels with disease severity. Thus, this strategy remains hypothetical and warrants further investigation.

#### 4.2.3. T-Cell Balance: Restoring Adaptive Immunity

Th17 cells secrete IL-17A, which promotes osteoclastogenesis [160]. Correcting the elevated Th17/Treg ratio is therefore pivotal [156]. Responsive materials restore this balance via zinc or magnesium ion release to favor Treg differentiation [158,161], targeted delivery of IL-17 antibodies or RORγt antagonists, and immunomodulatory cytokines (e.g., IL-10) or receptor antagonists (e.g., C5a receptor blockers) [32,162,163]. Restoring Th17/Treg equilibrium creates a pro-regenerative microenvironment. Furthermore, emerging evidence indicates that antioxidants (resveratrol, curcumin, N-acetylcysteine) and bioactive natural compounds such as baicalin can modulate immune cell metabolic reprogramming, contributing to Th17/Treg homeostasis restoration [164]. To date, no clinical trials have directly targeted the correction of the Th17/Treg ratio in periodontitis. Nevertheless, multiple studies have shown that changes in this ratio in the peripheral blood or tissues of patients can serve to assess its correlation with disease severity or as an indicator of treatment response [165].

### 4.3. Late-Stage Tissue Regeneration: Functional Remodeling

The ultimate goal is functional restoration of alveolar bone, cementum, and periodontal ligament. Late-stage regeneration requires materials to evolve from immunomodulatory shields to active regenerative scaffolds, integrating immuno-osteogenic coupling, angio-osteogenic synergy, stem cell homing, and epithelial exclusion.

#### 4.3.1. Immuno-Osteogenic Coupling

M2-polarized macrophages secrete osteoinductive cytokines, including bone morphogenetic protein-2 (BMP-2), transforming growth factor-β (TGF-β), and platelet-derived growth factor (PDGF). Natural compounds such as quercetin [75], ginsenoside Rg1 [139], and naringenin [62] have been incorporated into responsive carriers to sustain an anti-inflammatory niche. Biopolymer matrices such as chitosan and high-molecular-weight hyaluronic acid further enhance progenitor cell proliferation and migration [140,166]. Immunoosteogenic coupling has emerged as a cutting-edge direction in periodontal tissue regeneration, yielding substantial progress in basic research. Nevertheless, the translation from laboratory findings to clinical application requires a lengthy validation process, and this strategy currently remains at the preclinical research and development stage.

#### 4.3.2. Angio-Osteogenic Synergy

Effective bone regeneration requires concurrent neovascularization. Spatiotemporally programmed systems can achieve angio-osteogenic synergy [136]. Local statin delivery promotes angiogenesis and bone preservation [167], while alendronate incorporation into hydroxyapatite-hydrogel composites couples vascularization with osteoprogenitor adhesion [135]. A large body of preclinical studies has clearly demonstrated the potential of “vascular osteogenic coupling” for periodontal tissue regeneration, offering a robust theoretical and experimental foundation for mechanism-targeted therapeutic strategies [168].

#### 4.3.3. Stem Cell Homing and Multicellular Interactions

Modern biomaterials increasingly focus on endogenous cell homing. Cell-free platforms releasing SDF-1 recruit mesenchymal stem cells (MSCs) to defect sites, and thermosensitive hydrogels presenting SDF-1 further enhance MSC recruitment and osteogenic differentiation [137]. DPSC-derived exosomes deliver regulatory microRNAs that enhance host stem cell-mediated regeneration [72]. Extensive in vitro and in vivo studies have demonstrated that biochemical signals or scaffold materials can effectively recruit endogenous MSCs to periodontal defect sites and significantly promote periodontal tissue regeneration [169]. These findings provide strong support for a ‘cell-free’ tissue engineering strategy that circumvents the need for exogenous stem cell transplantation. Nevertheless, improving the recruitment efficiency and differentiation controllability of endogenous stem cells within the local microenvironment remains a critical challenge that hinders clinical translation.

#### 4.3.4. Space Maintenance and Epithelial Exclusion

Epithelial cells migrate faster than osteoblasts; without barrier materials, defects heal with non-functional epithelial tissue rather than bone. Advanced materials must therefore maintain defect space while creating a cell-occlusive barrier. Piezoelectric composite membranes generate microcurrents under masticatory stress, activating Piezo1 channels to transduce mechanical forces into osteogenic signals while simultaneously deterring epithelial ingrowth [67]. To date, several materials, including silk fibroin nanofiber membranes, have advanced to clinical trials [170].

### 4.4. Synergistic Integration: Diagnostic-Therapeutic Logic in All-in-One Platforms

Periodontitis involves temporally overlapping pathological processes. Current strategies increasingly integrate multiple therapeutic modalities within all-in-one carriers. For example, Dong et al. [141] developed a gelatin/oxidized dextran hydrogel co-loaded with calcium peroxide (CaO_2_) and penicillin. CaO_2_ generates local oxygen to reverse hypoxia and enhance penicillin efficacy against anaerobes. Advanced synergistic designs further integrate diagnostic logic with therapeutic delivery: stimuli-responsive elements detect pathological cues (pH, ROS, enzymes) and autonomously orchestrate the timing of drug release [171,172]. Among these strategies, oxygen-generating co-delivery systems have been validated in rodent models of periodontitis, demonstrating measurable reductions in anaerobic bacterial load and inflammatory markers [141]. Stimuli-responsive logic gating of drug release has likewise been demonstrated in vitro and in small-animal studies [171,172]. Fully autonomous closed-loop theranostic platforms, in which real-time biosensing continuously calibrates therapeutic output, however, remain conceptual at this stage and have not yet been realized in any periodontal application. Nevertheless, this field remains primarily at the proof-of-concept stage, with most studies still confined to in vitro or animal models. Challenges such as the need to verify long-term biosafety, achieve scalable production, and confirm therapeutic efficacy through rigorous clinical trials continue to hinder clinical translation.

## 5. Challenges and Future Perspectives

The rational design of microenvironment-responsive materials for periodontitis therapy rests on an integrated triad: versatile carrier platforms (e.g., hydrogels, microneedles, and vesicles), programmable responsive modalities, and spatiotemporal orchestration of therapeutic functions. By leveraging endogenous pathological signals (pH fluctuations, ROS, and bacteria-secreted enzymes), these strategies have demonstrated considerable promise in enabling rapid, on-demand therapeutic action while minimizing systemic adverse effects. To achieve effective intervention, these biomaterials must fulfill three essential roles: antibacterial activity, immunomodulation, and promotion of periodontal tissue regeneration. The development of multifunctional, synergistically integrated systems thus represents a highly promising direction. Nonetheless, significant challenges remain in bridging the gap between bench-top prototypes and clinical translation.

### 5.1. Multi-Stimulus Cascade Responsiveness

The periodontal lesion is characterized by a complex, overlapping matrix of stimuli (pathogenic colonization, localized hypoxia, acidosis, elevated ROS, dysregulated enzymes, and biochemical alterations such as increased ATP and lactate levels). A single responsive modality is often inadequate to address this complex milieu; consequently, the field is transitioning toward multi-stimulus cascade-responsive systems [173]. By integrating two or more responsive mechanisms via Boolean logic (e.g., AND gates), these platforms ensure that therapeutic release occurs only when multiple pathological criteria are met simultaneously or sequentially [174]. This cascade logic accommodates patient variability, lowers the response threshold at the target site, and enhances specificity [175]. Liu et al. [58] provided an exemplary demonstration by engineering a dual-responsive hydrogel. Following initial degradation by MMP-9 at the lesion site, the encapsulated metal–organic frameworks are exposed to the acidic microenvironment, providing the secondary stimulus necessary to trigger localized drug release.

### 5.2. Spatiotemporal Synchronization and Theranostic Integration

Despite encouraging preclinical outcomes, dynamic adaptation to evolving pathological conditions remains a significant challenge. Early-stage periodontitis necessitates aggressive antibacterial and antioxidant interventions, whereas later healing phases require pro-regenerative signals. Achieving precise spatiotemporal synchronization requires advanced structural engineering, exploiting differential bond cleavage kinetics to produce a temporally programmed profile with an early rapid burst followed by sustained late-phase release [176,177]. Additionally, integrating exogenous triggers such as photothermal stimulation enables clinicians to actively control secondary response events, facilitating early hemostasis followed by bone regeneration [178].

### 5.3. Clinical Translation and Biosafety Hurdles

Many reported systems face significant hurdles in clinical translation. Producing complex responsive nanozymes under Good Manufacturing Practice (GMP) conditions is particularly challenging, as batch-to-batch variability can compromise functional stability and safety [179]. Furthermore, conventional sterilization methods (high-pressure steam, gamma irradiation, or ethylene oxide) may alter stimuli-responsive chemical bonds, disrupt delicate microstructures, or cause premature drug leakage [180]. The unique anatomical constraints of the oral cavity further challenge material stability. Responsive carriers must withstand a dynamic fluidic environment and continuous mechanical forces from mastication and phonation, which impede precise targeted delivery [181]. Moreover, long-term biosafety investigations remain limited, particularly regarding systemic toxicological risks from degradation products and chronic material ingestion via saliva swallowing [182].

Despite notable advances in basic research on microenvironment-responsive materials for periodontitis, the overall clinical evidence remains immature. One major challenge is the uneven distribution of evidence across material types. Polymer microspheres and GTR fibrous membranes possess the most mature clinical evidence, substantiated by numerous randomized controlled trials. Hydrogels have accumulated preliminary clinical data, mainly from local drug delivery and antioxidant interventions. Evidence for liposomes and microneedles, although still limited, is growing rapidly. Conversely, clinical research on extracellular vesicles (EVs) and inorganic nanoparticles is extremely scarce and largely confined to proof-of-concept studies. For EVs, only a few such studies and retrospective case analyses exist; for instance, one study in stage I–III periodontitis demonstrated that EVs can significantly improve clinical parameters and reduce pro-inflammatory cytokines [183]. A second challenge is the hierarchical disparity in the clinical operability of different response mechanisms. pH-responsive and MMP-targeted strategies are backed by relatively extensive clinical biochemical data, offering a reliable basis for defining their response thresholds. Clinical evidence for ROS-responsive strategies stems largely from indirect validation through antioxidant approaches. Glucose-responsive systems have the weakest clinical foundation, as fundamental physiological data on the periodontal microenvironment in diabetic patients have yet to be systematically gathered. A third challenge is the absence of standardized clinical endpoint criteria. Efficacy indicators vary widely across studies, with some prioritizing clinical parameters such as probing depth and clinical attachment level, others emphasizing biochemical markers including MMP-8, inflammatory cytokines, or oxidative stress indices, and still others focusing on radiographic measures like bone defect fill. This heterogeneity severely impedes cross-study comparisons and meta-analyses. Key research gaps remain: first, large-sample, multicenter, randomized controlled trials with long-term follow-up are lacking; second, pharmacokinetic and pharmacodynamic data directly validating the intended stimuli-responsive behavior are absent, as current studies demonstrate efficacy but cannot confirm that the response mechanism functions as designed in vivo; third, subgroup analyses targeting populations most likely to benefit, such as diabetic patients, the elderly, and those with aggressive periodontitis, are insufficient; and fourth, systematic evaluations of long-term safety, in vivo degradation products, and potential nanotoxicity risks of these innovative materials remain scarce.

Simultaneously, within the context of microenvironment-responsive therapy for periodontitis, the local microenvironmental characteristics exhibit pronounced inter-individual variability [184], intra-individual heterogeneity across different tooth sites [185], and temporal fluctuations within the same diseased pocket, collectively manifesting as a highly dynamic state [44]. Such heterogeneity engenders substantial uncertainty in the therapeutic efficacy of microenvironment-responsive materials under diverse clinical conditions. For instance, although glucose-responsive systems can be designed for diabetic patients, the response may remain insufficient in individuals with severe local inflammation, whereas in well-controlled diabetic patients, the glucose concentration in gingival crevicular fluid may decline to near-normal levels, leading to a failure of the intended responsive design. Therefore, in patient-specific clinical scenarios, achieving precise “threshold matching” of the material to the local milieu is imperative, beyond merely enforcing rigorous standardization of the manufacturing process. However, the implementation of such precise matching encounters profound challenges. If the trigger threshold is calibrated against a patient’s personal biochemical baseline, this baseline itself is subject to gradual drift over time due to fluctuations in psychological status, lifestyle habits, acute events, aging, and the progression of chronic comorbidities, rendering its determination highly elusive. Furthermore, variations in biomarker levels induced by normal physiological processes may inadvertently provoke responsive release, undermining the therapeutic performance of the design. Accordingly, further exploration is warranted to attain accurate threshold control.

### 5.4. Future Roadmap

#### 5.4.1. Short-Term Expectations

To transition microenvironment-responsive materials from proof-of-concept prototypes to clinically viable tools, future research should prioritize the following directions:

- Disease timeline mimicry: designing multi-stimulus cascade-responsive materials with Boolean logic that mirror the biological trajectory of periodontal disease.
- Scalable manufacturing: overcoming GMP manufacturing and sterilization challenges through non-destructive processing technologies.
- Rigorous biosafety evaluation: conducting comprehensive large-animal and long-term safety studies to bridge the gap to clinical trials.

#### 5.4.2. Long-Term Expectations

The clinical implementation of microenvironment-responsive strategies still faces numerous limitations. In light of the current situation, a prolonged exploratory journey lies ahead for the following speculative development directions:

- Closed-loop platforms: integrating diagnostic sensing with targeted therapeutic action to create autonomous, adaptive theranostic systems. From a long-term perspective, theranostic device development represents a major future direction. By coupling biosensors for real-time microenvironmental monitoring with responsive delivery systems, such closed-loop platforms could autonomously calibrate therapeutic output to the evolving disease stage, enabling truly personalized and adaptive treatment.
- Personalized medicine: exploring patient-stratified approaches tailored to individual microenvironmental signatures and disease susceptibilities.

### 5.5. Application in Other Fields

Microenvironment-responsive materials also find broad utility in non-periodontal fields. Peri-implantitis similarly manifests a host inflammatory response and oxidative stress imbalance driven by biofilm dysregulation [186]. Fibrous membranes, microneedles, and inorganic nanoparticles have demonstrated considerable promise in oral implantology and bone regeneration [187]. Peri-implantitis-induced bone defects present a pronounced spatial heterogeneity that fundamentally challenges conventional therapeutic architectures: the outer soft-tissue-contacting region is enriched with pathogenic bacteria, inflammatory mediators, and acidic metabolites, demanding urgent anti-infective intervention, while the inner osseous defect requires strict sterility, a low-inflammatory microenvironment, and active promotion of bone regeneration. Single-phase, passive delivery scaffolds cannot reconcile these conflicting demands within a unified device. The integration of microenvironment-responsive fibrous membranes, microneedles, and inorganic nanoparticles into patient-specific grids or scaffolds, fabricated via advanced 3D printing, offers a pathway to embed responsive functionality directly into a defect-specific architecture and thereby orchestrate a truly personalized regenerative workflow. This integration exploits the complementary spatial and temporal advantages of each component: microneedles deliver responsive antimicrobial and immunomodulatory agents into the inflamed soft-tissue compartment on demand [188]; bilayered fibrous membranes simultaneously serve as physical barriers against epithelial ingrowth and as spatially resolved interfaces that support differential anti-inflammatory and osteogenic functions across the soft- and hard-tissue-facing surfaces [189]; and inorganic nanoparticles distributed throughout the scaffold matrix provide sustained catalytic antibacterial, antioxidant, and bone-promoting activity, with their optical sensing capabilities enabling longitudinal monitoring of therapeutic progression [190,191,192]. This paradigm represents a compelling direction for future translational research, though its clinical realization will require advances in manufacturing standardization, regulatory frameworks for combination devices, and long-term safety validation.

## Figures and Tables

**Table 1:** Summary of microenvironment-responsive mechanisms and chemical design strategies for periodontitis therapy.

Responsive Type	Material Platform	Responsive Chemistry/Mechanism	Pathological Trigger Context	Refs.
pH-responsive	Quaternary ammonium chitosan-liposome nanoparticle	Protonation; charge transition.	Localized Acidosis: Biofilm-induced acidic microenvironment (pH 5.5–6.5).	[19,61]
pH-responsive	Quaternized chitosan/oxidized dextran hydrogel	Dynamic Covalent Cleavage: Schiff base hydrolysis.	Localized Acidosis: Inflammatory metabolic shifts (pH 5.5–6.5).	[20]
pH-responsive	Carboxymethyl-hexanoyl chitosan	Charge Transition: Reversible protonation/deprotonation of carboxymethyl groups, disrupting inter-chain hydrogen bonding.	Localized Acidosis: Inflammatory metabolic shifts (pH 5.5–6.5).	[62]
ROS-responsive	Tri-thiol PBA ester-crosslinked PVA hydrogel	Oxidative Cleavage: Phenylboronic ester oxidation.	Severe Oxidative Stress: Neutrophil respiratory burst (e.g., H_2_O_2_ 50–200 µM).	[32]
ROS-responsive	Phenylboronic acid-modified PEI hydrogel	Oxidative Cleavage: ROS-triggered dissociation of phenylborate linkages within the dynamic hydrogel network.	Severe Oxidative Stress: Elevated ROS in the diabetic periodontal niche.	[33]
Enzyme-responsive (MMP)	Triglycerol monostearate/BHT lipid-like hydrogel	Enzymatic Hydrolysis: MMPs catalyze the specific hydrolysis of ester bonds within the lipid matrix.	MMP Overexpression: Elevated MMP-8 levels (>100 ng/mL) in deep periodontal pockets.	[12]
Enzyme-responsive (MMP)	MMP-2-cleavable peptide-crosslinked HA hydrogel	Peptide Cleavage: Specific enzymatic recognition and cleavage of the highly tailored GPLGVRG peptide sequence.	MMP Overexpression: Elevated MMP-2 activity driving tissue degradation.	[38]
Enzyme-responsive (ALP)	Polyphosphoester-crosslinked chitosan membrane	Enzymatic Hydrolysis: ALP catalyzes the nucleophilic attack and cleavage of P–O–P phosphoester bonds.	Biomarker Elevation: Significantly increased ALP activity (>1800 IU/L) in progressive periodontitis.	[42]
Enzyme-responsive (Gingipain)	Functional peptide-anchored PEG hydrogel	Pathogen-Specific Cleavage: Proteolytic cleavage of specific anchor sites uniquely recognized by the RgpA cysteine protease.	Microbial Dysbiosis: *P. gingivalis* colonization and active gingipain secretion.	[47]
Glucose-responsive	GOx/ZIF-8 core in calcium alginate shell	Enzymatic Cascade: GOx oxidizes glucose to gluconic acid, lowering pH and triggering acid-sensitive ZIF-8 structural collapse.	Hyperglycemia: Elevated local glucose (>7.0 mM) in diabetic periodontitis.	[50]
Glucose-responsive	PBA-functionalized alginate/chitosan hydrogel	Competitive Binding: Diols in glucose competitively bind to phenylboronic acid, shifting it to a hydrophilic tetrahedral state to induce swelling.	Hyperglycemia: Elevated local glucose (>7.0 mM) in diabetic periodontitis.	[52]

**Table 2:** Overview of material platforms for periodontitis therapy.

Material Type	Key Advantages	Clinical Limitations	Representative Functional Design Highlights	Refs.
Hydrogels	Injectability; biomimetic ECM-like structure; high drug loading.	Weak mechanical strength; risk of physiological washout.	pH-responsive Schiff base, Embelin release	[63]
Microneedles (Conventional and Core–shell)	High mucosal permeability; minimally invasive; overcomes epithelial barrier.	Uncertain long-term release kinetics; limited chronic biosafety data.	Wet-adhesive MNs; core–shell dual release	[64,65]
Fiber Membranes (Conventional and Janus)	Surgical GTR barrier; sustained space maintenance.	Limited multifunctionality; risk of epithelial ingrowth.	Immunomodulatory [66]; piezoelectric Janus membrane [67]	[66,67]
Polymer Microspheres (Adhesive and Biomimetic)	Site-specific administration; tunable prolonged retention.	Susceptibility to GCF flushing; initial burst release.	Adhesive microspheres [68]; biomimetic sericin/HA [69]	[68,69]
Inorganic Nanoparticles (Nanozyme and MBG)	Intrinsic bioactivity; rigid architecture; multimodal functions.	Potential cytotoxicity; rigid structure limits large cargo.	Cu_x_O nanoparticles (miRNA, nanozyme) [70]; MBG (Si, Ca) [71]	[70,71]
Vesicles (Exosome and Liposome)	Subcellular membrane fusion; low immunogenicity; biomimicry.	Exosomes: high cost and isolation complexity. Liposomes: structural fragility.	DPSC exosomes [72]; liposome-in-hydrogel [73]	[72,73]

**Table 3:** Spatiotemporal orchestration of microenvironment-responsive functions: from early stabilization to late-stage regeneration.

Functional Phase	Active Component	Carrier Platform	Key Functional and Mechanistic Highlights	Refs.
Early-to-Mid Phase: Microecological Remodeling and Immunomodulation	Tetracycline/ZnO	PLGA MS/PCL Fiber	Targeted release; GTR barrier function; bioadhesion	[127,128]
Early-to-Mid Phase: Microecological Remodeling and Immunomodulation	Chlorhexidine acetate	HA/GelMA Hydrogel	Self-healing; antibacterial; antioxidant; anti-resorptive	[129]
Early-to-Mid Phase: Microecological Remodeling and Immunomodulation	Apoptotic EVs/Quercetin	GelMA/Poloxamer	Neutrophil apoptosis induction; M2 polarization; Th17/Treg rebalancing	[130,131]
Early-to-Mid Phase: Microecological Remodeling and Immunomodulation	8-Aminoguanosine	Boronic acid Hydrogel	MAPK/NF-κB modulation, ROS inhibition, M1 suppression	[132]
Early-to-Mid Phase: Microecological Remodeling and Immunomodulation	Copper tannic acid/DTT	TGM/PEGDA Hydrogel	Nanozyme-mediated ROS scavenging; pro-healing	[12,47]
Early-to-Mid Phase: Microecological Remodeling and Immunomodulation	Chrysin/Caffeic acid	CMCS Hydrogel	Antioxidant, immunomodulatory, pro-osteogenic	[133]
Early-to-Mid Phase: Microecological Remodeling and Immunomodulation	Metformin	Copolymer Hydrogel	AMPK/β-catenin, reverses hyperglycemia, recruits BMSCs	[134]
Late-Phase: Tissue Regeneration and Functional Restoration	Alendronate/HA/NapGFF	Composite Matrix	Anti-osteoclastic activity; bone reconstruction	[135]
Late-Phase: Tissue Regeneration and Functional Restoration	SDF-1/BMPs	Nap-FFY/PEGDA	MSC homing; antimicrobial activity; osteogenic differentiation	[47,136]
Late-Phase: Tissue Regeneration and Functional Restoration	PDLSCs/iPSCs	Chitosan/Gelatin	Growth factor delivery; anti-apoptotic; pro-angiogenic	[137,138]
Late-Phase: Tissue Regeneration and Functional Restoration	Ginsenoside Rg1/Ca-Aluminate	HA-Chitosan/Chitosan	Biomineralization; osteogenic gene upregulation; bone loss reduction	[139,140]
Synergistic Integration	CaO_2_/Penicillin	Dextran-Gelatin	In situ O_2_ generation enhancing antibiotic efficacy	[141]

Note: Early- and mid-stage therapeutic functions (e.g., microbial control and osteoimmune reprogramming) often exhibit significant temporal overlap and functional synergy; they are therefore categorized together as the stabilization phase to reflect the continuous nature of inflammation resolution.

## Data Availability

No new data were created or analyzed in this study. Data sharing is not applicable to this article.
